# *Trans*-acting proteins regulating mRNA maturation, stability and translation in trypanosomatids

**DOI:** 10.1016/j.pt.2010.06.011

**Published:** 2011-01

**Authors:** Susanne Kramer, Mark Carrington

**Affiliations:** Department of Biochemistry, University of Cambridge, Tennis Court Road, Cambridge, UK, CB2 1QW

## Abstract

In trypanosomatids, alterations in gene expression in response to intrinsic or extrinsic signals are achieved through post-transcriptional mechanisms. In the last 20 years, research has concentrated on defining the responsible *cis*-elements in the untranslated regions of several regulated mRNAs. More recently, the focus has shifted towards the identification of RNA-binding proteins that act as *trans*-acting factors. Trypanosomatids have a large number of predicted RNA-binding proteins of which the vast majority have no orthologues in other eukaryotes. Several RNA-binding proteins have been shown to bind and/or regulate the expression of a group of mRNAs that code for functionally related proteins, indicating the possible presence of co-regulated mRNA cohorts.

## Regulation of gene expression in trypanosomatids

Trypanosomatids, including the pathogens from the genera *Trypanosoma*, *Leishmania* and *Crithidia,* separated early in evolution from other eukaryotes and possess several unusual features in their biology. One is genome organization and transcription. Each chromosome has clusters of tandemly arranged protein coding genes that are co-transcribed by RNA polymerase II from an unknown promoter at the 5′-end of the array [1]. In most cases the genes in an array have no obvious functional relationship. Mature mRNAs arise through a *trans*-splicing reaction that adds a capped exon (spliced leader) of 39 nucleotides to the 5′-end and a coupled process that both polyadenylates the pre-mRNA and cleaves it from the upstream gene [2–4]. The only RNA polymerase II promoter that has been characterized drives spliced leader RNA transcription [5–7]. It was recently shown that distinct epigenetic modifications are present at the putative start and endpoints of other RNA Polymerase II transcription units and these may be central to regulating transcription for protein coding genes [8]. All evidence indicates that transcription of all protein coding genes by RNA polymerase II occurs at similar rates. Nevertheless, huge differences in expression levels are found between different genes or for the same gene in different conditions. For example, *T. brucei* transcriptome analyses report between 5 and 25% of mRNAs to be differentially regulated between two life-cycle stages [9–13].

Two mechanisms have evolved for very high expression levels: (i) gene copy number increase resulting in tandem arrays (ii) transcription by RNA polymerase I of a few genes encoding mainly the super-abundant cell surface proteins [14]. The majority of protein coding genes are single copy and controlled by one or more of the following: efficiency of pre-mRNA maturation, mRNA half-life, translational efficiency and protein half-life [15]. The dependence on post-transcriptional regulation of a whole genome is unprecedented. The regulation of individual genes is likely to be a combinatorial mechanism based on the interaction of *trans*-acting factors with *cis*-elements in each mRNA. The majority of *cis*-acting elements affecting mRNA half-life and translation have been detected in 3′-untranslated regions (UTRs). More recently, research has concentrated on *trans*-acting factors that interact with these *cis*-elements, and herein, studies specifically focusing on RNA-binding proteins that either have been shown to play a role in post-transcriptional regulation or have the potential to regulate based on the function of related proteins in other organisms are discussed. The *trans*-acting factors are ordered on the types of structural domains involved in RNA-binding: RRM domain containing proteins, CCCH zinc finger proteins and PUF proteins. Proteins solely dependent on other motifs for RNA-binding, such as the K homology domain (KH) (only two proteins are predicted in the genome), the arginine glycine rich motif (RGG) or the cold shock domain (CSD), have not been characterised to date.

## RNA-binding proteins containing RRM domains

The RNA recognition motif (RRM) is the most abundant RNA-binding motif in eukaryotes [16]. It consists of approximately 90 amino acids with a βαββαβ topology. The β-strands form a four-stranded β sheet that is the main RNA-binding element. The binding abilities of RRM motifs are very diverse; this motif can bind between two and eight nucleotides of single stranded RNA with very different affinities and specificities, but also other proteins including other RRM domains. Sequence specificity of RNA binding is achieved slightly differently for each RRM and it is not yet possible to predict the RNA substrate for a given RRM. Diverse RRM-type proteins regulate gene expression by controlling splicing, mRNA stability or translation.

Kinetoplastids have over 75 RRM domain containing proteins [17]. Only a few (between 13 and 21) have readily recognizable orthologues in other eukaryotes, including the poly(A)-binding proteins, the translation initiation factor EIF3B and the splicing factor U2AF35. Five of the proteins, all unique to kinetoplastids, have been experimentally characterized and all were found to play a role in regulating mRNA stability: (i) three single RRM domain proteins (UBP1, UBP2, RBP3), (ii) one double RRM domain protein (DRBD3/PTB1) and (iii) one protein with four RRM domains (DRBD4/PTB2).

The very first *trans*-acting factor identified in kinetoplastids was *Tc*UBP1 (*T. cruzi* uridine binding protein 1). The starting point for its identification was the discovery of AU-rich elements (AREs) in the 3′-UTRs of the developmentally regulated *T. cruzi* small mucin mRNAs (*TcSMUG*) [18]. AREs are common regulatory elements of short-lived eukaryotic mRNAs [19], and the pathway appears to be conserved, since overexpression of the mammalian ARE-stabilizing protein HuR stabilized an ARE-containing mRNA in *T. brucei* [20]. The AREs were shown to mediate developmental regulation of the *TcSMUG* mRNA by destabilization in the metacyclic but not the epimastigote stage [18]. *Tc*UBP1 binds to the ARE of the *TcSMUG* mRNA *in vitro*, and overexpression destabilizes *TcSMUG* mRNA *in vivo*, suggesting it acts as a destabilizing factor [21]. The protein is five-fold downregulated in the epimastigote stage compared to the metacyclic stage, providing a possible explanation for the stability of the *TcSMUG* mRNA in epimastigotes [21]. TcUBP1 can homodimerize and is present in a large ribonucleoprotein complex, together with TcUBP2 (which is similar to TcUBP1, but has different N- and C-termini and reverse developmental regulation) and poly(A)-binding protein 1 [22]. Binding to poly(A)-binding protein prevents TcUBP1 dimerization, and it has been speculated that this can decrease the affinity of the poly(A)-binding protein for the poly(A) tail, thus enhancing mRNA degradation [22]. In addition to *TcSMUG*, 39 other transcripts were found bound to TcUBP1 by coprecipitation [23]. One *cis*-acting element, a stem-loop structure, has been identified in the 3′-UTRs of the majority of these TcUBP1 target mRNAs. It was 16-fold over-represented in these mRNAs in comparison to the full genome and was responsible for the binding to TcUBP1 [23]. Moreover, this *cis*-element was successfully used for the prediction of novel UBP1 target mRNAs [23]. Nevertheless, it was not present in all UBP1 targets, and it was different from the previously identified ARE, indicating the involvement of more than one *cis*-element [23]. Both UBP1 and UBP2 proteins are mainly localized in the cytoplasm, but also localize to stress granules upon starvation and to the nucleus under arsenite stress; for TcUBP1 this localisation has been shown to be dependent on the RRM domain [24,25].

Similar experiments have been performed with the *T. brucei* orthologues of UBP1 and UBP2. Overexpression or RNAi depletion of *Tb*UBP1 and *Tb*UBP2 was lethal and had selective effects on the levels of a group of mRNAs, including mRNAs coding for a family of F-box proteins [26]. Many F-box proteins are components of the SCF E3 ubiquitin ligase complex involved in cell cycle control. A family of transmembrane proteins of unknown function were differentially affected by UBP1/2 RNAi, some increased and some decreased in level [27]. The UBP targets identified in *T. brucei* are different than the ones identified in *T. cruzi*, and it remains to be seen whether this reflects a real difference between the organisms or is due to a difference in experimental approach.

The third protein containing a single RRM domain studied in trypanosomes is RBP3. TcRBP3 has different RNA-binding specificities than TcUBP1/2 as judged by homopolymer binding assays [28] and it binds a different set of mRNAs, which mainly encode ribosomal proteins; only a few targets overlap between TcRBP3 and TcUBP1 [23]. A *cis*-element forming a stem-loop was identified in RBP3 targets, but the sequence was unlike that of the *cis*-element present in the TcUBP1 target mRNAs [23]. Five mRNAs coprecipitated with the *T. brucei* orthologue of RBP3; two coded for CCCH proteins (ZFP1; ZC3H11), one for the F-box protein CFB1 and two for proteins with unknown function [29].

A second ARE that was used for the identification of *trans*-acting factors was found in the 3′-UTR of the developmentally regulated *T. brucei* phosphoglycerate kinase B (*PGK-B*) mRNA [20]. This ARE destabilized *PGK-B* mRNA in the bloodstream stage and was used as bait for *trans*-acting factors. Four proteins, DRBD3, RBP33, Tb927.6.4440 and UBP2, were identified as binding to the element. [30]; however, the one protein further characterized, the double RRM domain-containing protein DRBD3, did not bind to *PGK-B* mRNA. RNAi knock-down of DRBD3 destabilized a subset of 21 mRNAs that were enriched for mRNAs coding for developmentally regulated transmembrane proteins, indicating a function of DRBD3 as a stabilizer of this subset of mRNAs [30]. DRBD3 was found to bind to most of the 21 mRNAs, but the *cis*-element remains elusive. The UAUUUUUU element identified as over-represented in mRNAs upregulated in the procyclic stage [31] was present in all mRNAs up-regulated after DRBD3 knock down, but was also present in many mRNAs unaffected by the knockdown. DRBD3 is an essential protein that is not developmentally regulated and localizes to the cytoplasm and the nucleus [30]. In a subsequent study, DRBD3 and the four-RRM domain-containing protein DRBD4 were renamed PTB1 and PTB2, based on limited identity to human polypyrimidine tract binding proteins [32]. RNAi depletion of PTB1 caused a decrease in mRNA levels of many transcripts [32], and there was overlap with the mRNAs identified previously [31]. Knockdown of PTB2 affected a different set of transcripts. PTB1 and PTB2 were shown to be essential for *trans-*splicing of a subset of genes containing C-rich polypyrimidine tracts, evidence for a function in splicing similar to their mammalian counterparts [32].

## RNA-binding proteins containing CCCH motifs

The CCCH type zinc finger proteins, defined by the C-X_4-15_-C-X_4-6_-C-X_3_-H zinc finger motif [33,34], bind nearly exclusively to single-stranded RNA and play regulatory roles in all processes of mRNA metabolism. The best studied CCCH type zinc finger proteins are of the TIS-11 family, with the most prominent member being the mammalian protein tristetraprolin (TTP) (reviewed in [35]). TIS-11 proteins bind to AU-rich regions in the 3′-UTRs of their targets and cause deadenylation and, in most cases, mRNA degradation. It is not clear how sequence specificity of CCCH proteins for target mRNAs is achieved.

The genomes of *T. brucei*, *T. cruzi* and *Leishmania* contain 48, 51 and 54 non-redundant CCCH proteins, respectively [36,37]. One third of these have more than one CCCH motif allowing possible multivalent RNA-binding, and one third have additional identifiable domains [36]. Only five of the kinetoplastid CCCH proteins have overall orthology to proteins in other organisms: (i) the splicing factor U2AF35 [38], (ii) two components of the cleavage and polyadenylation apparatus (CPSF30 and FIP1) [39,40], (iii) the putative homologue of the mRNA export factor MEX67 [36], and (iv) a 3′ to 5′ exoribonuclease [36]. Interestingly, a CCCH motif is not found in Mex67 in other organisms, and the combination of CCCH domain and 3′ to 5′ exoribonuclease is unique to *Leishmania*, indicating differences in the mechanisms of function of these proteins to their orthologues in other eukaryotes.

**Figure 1:**
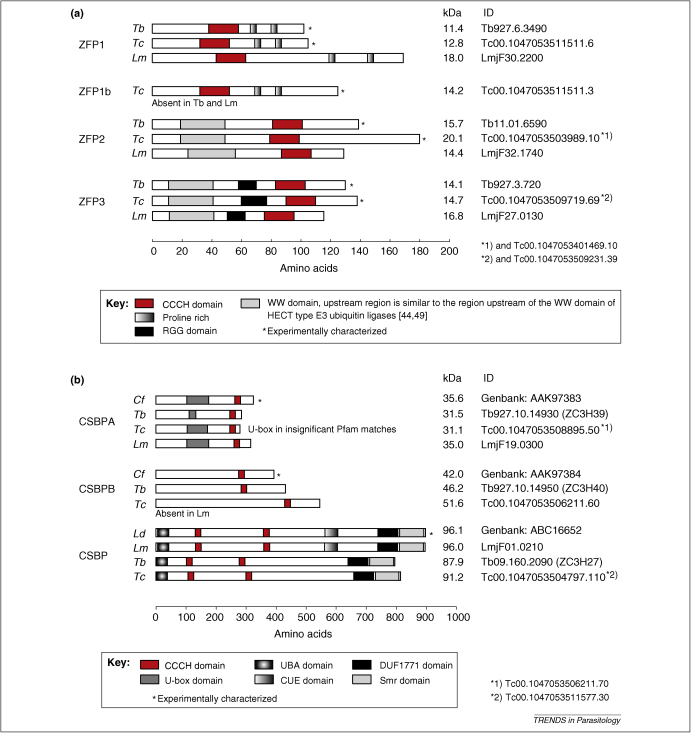
Schematics of all CCCH-type zinc finger proteins with at least one trypanosome orthologue being experimentally characterized. **(a)** The small ZFP family of CCCH proteins involved in the regulation of differentiation. **(b)** The cycling sequence binding proteins, CCCH proteins that bind to an octameric sequence present in mRNAs that change level during the cell cycle. The *T. cruzi* genome strain CL Brener is a hybrid and allelic alleles have been indicated (*). Abbreviations: *Tb, T. brucei; Lm, L. major; Ld, L. donovani; Tc, T. cruzi; Cf, Crithidia fasciculata*.

Of the CCCH proteins unique to kinetoplastids, two families have been experimentally characterized: the small ZFP proteins involved in the regulation of differentation, and the cycle sequence binding proteins (CSBP) that bind to a conserved sequence in mRNAs that have increased expression levels in late G1 and S-phase. In *T. brucei*, there are three ZFP proteins each with a single CCCH domain, ZFP1 (ZPF1A), ZFP2 (ZPF2A), and ZFP3 (ZPF2B). *T. cruzi* has one additional ZFP1 homologue, ZFP1B [41]. In addition to CCCH domains, ZFP1s have two proline-rich regions; ZFP2 and ZFP3 have WW domains that bind to proline-rich regions [42], and ZFP3 also has an RGG motif, which often contributes to the assembly of RNP complexes (reviewed in [43]) (Figure 1(a)). The presence of the WW motif in ZFP2 and ZFP3 and the proline-rich regions in ZFP1 suggested the formation of ZFP complexes, and WW-motif dependent interactions between ZFP1 and ZFP2/ZFP3 have been shown in both *T. cruzi* and *T. brucei* [41,44]. No interactions between ZFP2 and ZFP3 were detected in yeast 2-hybrid assays, but ZFP2 was co-immunoprecipitated with ZFP3, suggesting the presence of ZFP proteins in larger complexes [44]. Such complex formation suggests a possible way to achieve higher affinity RNA-binding through multivalency. Recombinant *T. cruzi* ZFP1 has been assayed for binding to different oligonucleotides. The highest affinity binding was to poly-C and CU-rich oligonucleotides rather than poly A, GU-rich or ARE oligonucleotides [45]. It did, however, bind to the AU-rich 26-mer element present in the 3′-UTR of the developmentally regulated *T. brucei EP*/*GPEET* mRNAs, which encode procyclins, the predominant cell surface protein expressed in procyclic forms, but these complexes were less stable than the complex formed with a poly-C oligomer [45]. This 26-mer element is necessary, but not sufficient, to decrease mRNA stability and translation of the *EP/GPEET* mRNA in the bloodstream form stage [46]. Binding of ZFP1 to the 26-mer could be competed by a two-fold excess of unlabeled 26-mer elements, but not by a 100-fold excess of control ARE oligonucleotides [45]. In this context it is interesting that ZFP3 binds to *EP1/GPEET* mRNAs [44,47], raising the possibility of the entire ZFP complex being involved in the regulation of stability and/or translation of the *EP/GPEET* mRNAs.

Alteration of expression of each of the three ZFPs interfered with differentiation from bloodstream into procyclic cells: deletion of the *Tb*ZFP1 gene impaired kinetoplast positioning during differentiation [48], cells depleted of *Tb*ZFP2 by RNAi were unable to differentiate [49], and *Tb*ZFP3 overexpression caused an enhanced rate of differentiation [44]. The role of ZFPs is not restricted to differentiation. In established procyclic cell lines overexpression of both ZFP2 and ZFP3 caused a gross extension of the microtubule cytoskeleton at the posterior pole, termed the nozzle phenotype [44,49]. ZFP3 binds the *EP1* and *GPEET* mRNAs. This interaction is dependent on the presence of two well characterized *cis*-elements in the procyclin 3′-UTR, among it loop II, which includes the 26-mer discussed above, as well as on the CCCH motif of ZFP3 [44,47]. Interestingly, while ZFP3 overexpression causes no change in *EP1* and *GPEET* mRNA levels, an increase in EP1 protein and a decrease in GPEET protein was observed, providing strong evidence that ZFP3 acts as a regulator of translation [47]. This role in regulating procyclin expression is reinforced by the finding that ZFP3 is polysome-associated in procyclic but not in bloodstream form cells [44]. Taken together, the data provide strong evidence for the ZFPs being involved in the regulation of differentiation processes and, for at least ZFP3, being a regulator of translation.

Three proteins with CCCH domains, cycling sequence binding proteins (CSBP), were found to bind mRNAs that fluctuate during the cell cycle. CSBPA and CSBPB were identified in *Crithidia fasciculata* as binding mRNAs expressed during S-phase. These mRNAs included those coding for the kinetoplast associated type II DNA topoisomerase TOP2, the large subunit of the nuclear replication protein-A RPA1, the kinetoplast histone H1-like DNA binding protein KAP3 and the dihydrofolate reductase-thymidylate synthase DHFR-TS [50]. For *TOP2* and *RPA1*, sequences in the 5′-UTR were both necessary and sufficient for the cycling [51,52]; for *KAP3*, cycling elements were located in both the 3′-UTR and the intergenic regions, thus, cycling of at least *KAP3* must be controlled prior to mRNA maturation [53]. All sequences contained two or more conserved octamers, CAUAGAAG (or very similar), that were necessary but not sufficient for cycling [51,52]. Interestingly, longer sequences sufficient for cycling could still mediate mRNA cycling when transferred from the 5′-UTR to the 3′-UTR in the correct orientation [52], indicating that the location of the cycling element in the mRNA did not restrict function. The CCCH proteins CSBPA and CSBPB (Figure 1(b)) were purified by RNA affinity to the conserved octamer sequences [54,55]. However, knockout of CSBPA, which resulted in the absence of both CSBPA and CSBPB protein, showed that neither was necessary for mRNA cycling [56]. Three further proteins were identified to bind the conserved octamer: RBP63 (poly(A)-binding protein 2), RBP45 and RBP33 [57]. None of these proteins has a CCCH motif, and RBP45 and RBP33 have no recognizable RNA-binding domain. It remains unclear whether these proteins are necessary for mRNA cycling. In *Leishmania donovani*, a completely different CSBP protein, *Ld*CSBP, binds the CAUAGAAG octameric cycling sequence [58]. *Ld*CSBP has two CCCH domains, as well as two ubiquitin interacting domains (UBA and CUE) and is ubiquitinylated (Figure 1(b)). To conclude, all three CSBP proteins have been shown to bind to the mRNA sequence that is necessary for the cell cycle dependent changes in mRNA levels; however, CSBPA and CSBPB are not required for the cycling of the mRNA and their functions remain unclear.

## RNA-binding proteins containing PUF domains

Puf proteins, named after their founding members Pumilio (*D. melanogaster*) and Fem-3-binding factor (*Caenorhabditis elegans*) [59,60], regulate mRNA expression by binding to specific sequences in 3′-UTRs and recruiting proteins that then mediate mRNA degradation or translational repression (reviewed in [61]). Strikingly, and in contrast to most other RNA-binding proteins, RNA sequence recognition by Puf proteins can be predicted to some extent, [62,63] and in some cases has allowed manipulation of Puf protein sequence to alter the preferred binding sequence [64]. mRNAs regulated by the same Puf protein are often functionally related and thus constitute post-transcriptional RNA cohorts. For instance, yeast Puf1 and Puf2 bind preferentially to mRNAs encoding membrane-associated proteins [65] and the targets of human Pum1 are enriched for mRNAs coding for proteins involved in the regulation of transcription, cell cycle and proliferation [66]. Trypanosomes and *Leishmania* contain at least ten Puf proteins [67,68], more than *S. cerevisiae* (six), *Drosophila* (one), or humans (two).

Ten *T. cruzi* Puf proteins have been analysed and grouped according to the likelihood that they would bind the canonical Puf binding site: the 32 bp nanos response element (NRE) present in the 3′-UTR of the Puf-regulated *Drosophila* hunchback mRNA [59]. Group 1 (TcPUF1, TcPUF2) was predicted to bind the entire NRE sequence [67]. Group 2 (TcPUFs3-6, TcPUF9) was predicted to bind at least one conserved motif within the NRE sequence, whereas the PUF proteins of group 3 (TcPUFs7-8, TcPUF10) were more diverse and unlikely to bind NRE [67]. Interactions of TcPUF1 and TcPUF6 with NRE have been experimentally confirmed [67,69]. An RNAi screen of individual and some pairs of *T. brucei* PUF proteins in both cultured bloodstream forms and procyclics did not initially produce any growth effects [68], but in subsequent experiments RNAi depletion of TbPUF7 and TbPUF9 caused a reduction in growth rate [70,71]. The contrasting results are possibly because of differences in the level of protein knockdown between the experiments. To date, three PUF proteins have been studied in greater detail: TbPUF1/TcPUF6 (note the different nomenclature between the orthologues!), TbPUF9 and TbPUF7.

TbPUF1 was originally identified as a factor interacting with ESAG8, a putative regulatory protein accumulated in the nucleolus [72]. A TbPUF1 null (−/−) procyclic cell line was viable, and no significant changes of the transcriptome or proteome were found following RNAi depletion of TbPUF1 in either procyclic or bloodstream form stages [68]. In *T. cruzi*, a three- to five-fold overexpression of the orthologous TcPUF6 had no effect on growth or differentation but the transcriptome was clearly affected; 232 genes were more than three-fold upregulated, and 37 were more than three-fold downregulated [73]. Precipitation of TcPUF6 by tandem affinity purification identified eight mRNAs. All were among the genes that were significantly downregulated following *Tc*PUF6 overexpression, indicating that PUF6 destabilizes its mRNA targets. Six out of seven tested PUF targets were developmentally regulated. Interestingly, *Tc*PUF6 co-immunoprecipitates the P-body marker DHH1 [73], a protein recently found to be involved in regulating developmental gene expression in *T. brucei* [74].

Four *T. brucei* mRNAs were found to coprecipitate with a TAP tagged PUF9, coding for a kDNA ligase (LIGKA), a histone H4 variant and two unknown proteins, named PUF nine target 1 (PNT1) and PUF nine target 2 (PNT2) [70], the latter being identical to the previously described chromosome passenger protein TbCPC2 [75]. Of the four mRNAs, three (*LIGKA*, *PNT1* and *CPC2*) were stabilized by PUF9 overexpression and destabilized by PUF9 RNAi depletion, suggesting that PUF9, unlike any other known PUF protein, stabilizes its target mRNAs; histone H4 mRNA was largely unaffected. Interestingly, the *LIGKA*, *PNT1* and *CPC2* mRNAs showed cell cycle dependent fluctuations in mRNA levels with a peak in S-phase, and these fluctuations were removed by RNAi knockdown of PUF9, indicating that PUF9 might stabilize its target mRNAs specifically in S-phase. It is possible that all three PUF9 target mRNAs code for proteins with a regulatory function in the cell cycle; both LIGKA and PNT1 localize to the kinetoplast, and interference with their expression levels causes defects in kinetoplast replication [70,76,77]. CPC2 was copurified with the *T. brucei* orthologue of protein kinase Aurora B [75], localizes to the mitotic spindle in metaphase and early anaphase [70,75], and RNAi depletion causes inhibition of spindle assembly, chromosome segregation and cytokinesis [75]. The PUF9 target mRNAs might represent a post-transcriptional RNA cohort with a regulatory function in cell cycle, but further experimental evidence will be needed.

More recently, it has been reported that RNAi depletion of TbPUF7, which localizes to the nucleolus, causes a reduction in growth rate and inhibition of pre-ribosomal RNA processing [71].

## Conclusions

In the last few years, several proteins have been identified in trypanosomatids that regulate either mRNA stability or translation. Many interact with or regulate a specific subset of functionally related mRNAs, providing evidence for the existence of coregulated mRNA cohorts that have recently become a very popular model for the post-transcriptional regulation [65,78]. A summary is shown in Table 1. The presence of groups of coregulated mRNAs is also favoured by transcriptome studies of differentiation processes of *T. brucei* and *L. donovani* that found coregulated mRNAs often being enriched for mRNAs coding for proteins of the same pathway [10,11,79]. Nonetheless, post-transcriptional regulation of a given gene will not be based on one single *trans*-acting factor in most cases, but will be dependent on a combination of such factors either competing for the same *cis*-acting elements or binding to a different one; it is unlikely that any two different mRNAs are regulated by exactly the same set of *trans*-factors. The presence of several additional levels of post-transcriptional control, such as mRNA splicing, mRNA nuclear export, as well as protein half-life and protein modification, suggests that overall regulation is more complex. Last but not least, the most interesting observation about the RNA-binding proteins in trypanosomatids is the fact that most of them have no readily identifiable orthologues in other eukaryotes, providing considerable potential to discover novel, unusual pathways. Only a small fraction of these proteins has been experimentally analysed to date.

## Figures and Tables

**Table 1:** Summary of *trans*-acting factors in trypanosomes

*trans*-acting factor	target mRNAs					Ref.
	specific mRNA	group of mRNAs (enriched features)	*cis*-acting element (not necessarily sufficient)	effect of *trans*-acting factor on targets	binding to target mRNAs shown	
TcUBP1	small mucin *SMUG*		AU rich element	mRNA destabilization	yes	[21]
TcUBP1		glycoproteins, metabolism	stem loop	N.D.^a^	yes	[23]
TbUBP2	F-box protein (*CFB1*)		3′-UTR	mRNA stabilization	yes	[26]
TbUBP1/2	transmembrane protein family		3′-UTR	mRNA stabilization and mRNA destabilization	no	[27]
TcRBP3		ribosomal proteins	stem loop	N.D.	yes	[23]
TbRBP3	ZFP1; ZC3H11, CFB1 Tb927.4.1000 and Tb927.8.7820			no significant effects seen	yes	[29]
TbDRBD3		developmentally regulated / transmembrane	U-rich elements	mRNA stabilization	yes	[30]
TbDRBD3 =PTB1		mRNAs with C-rich polypyrimidine tracts in 5′UTR	N.D.	mRNA stability, *trans*-splicing	yes	[32]
TbDRBD4 =PTB2		mRNAs with C-rich polypyrimidine tracts in 5′UTR	N.D.	mRNA stability, *trans*-splicing	no	[32]
TbZFP3	*EP1*		loop II and loop III of 3′-UTR	increase in translation	yes	[44,47]
TbZFP3	*GPEET*		N.D.	decrease in translation	yes	[44,47]
TcPUF6		developmentally regulated	N.D.	mRNA destabilization	yes	[73]
TbPUF9	LIGKA, PNT1, PNT2=CPC2	possible function in cell cycle	N.D.	mRNA stabilization	yes	[70]
LdCSBP	N.D.	N.D.	CAUAGAAG	N.D.		[58]
CfCSBPA/B	*TOP2, RPA1, KAP3, DHFR-TS*	specifically expressed during S-phase	CAUAGAAG	not necessary for cycling of target mRNAs	yes	[51–56]

a N.D. = Not determined.
